# Form-stable phase change composites based on nanofibrillated cellulose/polydopamine hybrid aerogels with extremely high energy storage density and improved photothermal conversion efficiency

**DOI:** 10.1039/d0ra10485j

**Published:** 2021-02-02

**Authors:** Yunlong Tan, Xiaosheng Du, Zongliang Du, Haibo Wang, Xu Cheng

**Affiliations:** College of Biomass Science and Engineering, Sichuan University Chengdu 610065 China scuchx@163.com +86-28-85401296; The Key Laboratory of Leather Chemistry and Engineering of Ministry of Education, Sichuan University Chengdu 610065 PR China

## Abstract

The development of form-stable phase change materials (PCMs) with superior photothermal conversion efficiency and high phase change enthalpy is critical for the utilization of solar energy. In this work, nanofibrillated cellulose (NFC)/polydopamine (PDA) hybrid aerogels (NPAs) were synthesized by cation-induced gelation of NFC/PDA suspension. Then, novel form-stable PCMs with superior energy storage density and improved photothermal conversion efficiency were successfully synthesized by impregnating *n*-octacosane into NPAs. Differential scanning calorimetry (DSC) analysis showed that the composite PCMs exhibited extremely high phase transition enthalpy (>248 J g^−1^) and excellent thermal reliability. Thermogravimetric analysis (TG) showed that the composite PCMs exhibited excellent thermal stability. In photothermal experiments, PDA acted as a photon trap and effectively improved the photothermal conversion efficiency (up to 86.7%) of the composite PCMs. In conclusion, the synthesized composite PCMs displayed high phase change enthalpy and superior photothermal conversion efficiency, suggesting their promising characteristics for solar energy utilization applications.

## Abstract 2

Novel form-stable composite phase change materials were fabricated by impregnating *n*-octacosane into nanofibrillated cellulose/polydopamine hybrid aerogels.

## Graphical abstract

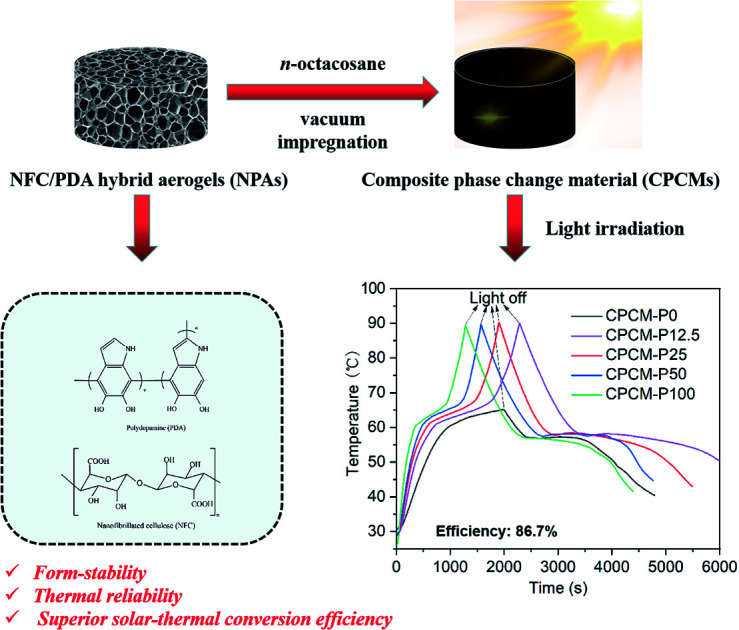

## Introduction

In recent years, a serious energy crisis and climate problems have occurred because of the over-exploitation of fossil fuels.^1–4^ Solar energy, as the most clean and inexhaustible renewable resource, has drawn intensive attention.^5–7^ However, the intrinsic properties of solar energy, including low energy density, intermittency, and inefficiency, limit its large-scale application. An effective method to overcome the intermittency of sunlight and improve the solar energy utilization efficiency is to develop thermal energy storage (TES) systems based on phase change materials.^8–10^

The phase change materials (PCMs) can be used to store and release large amounts of energy with little temperature change. Numerous PCMs, including organic PCMs (such as *n*-alkanes, carboxylic acid, and polyols) and inorganic PCMs (such as crystalline hydrated salts, molten salts, and metal alloys), have been researched for various fields of solar collectors, energy-saving buildings, thermal management of textiles, waste heat recovery.^11–17^ Among them, *n*-alkanes has attracted attention of researchers owning to its non-toxicity, high energy storage capacity, suitable phase change temperature and chemical stability.^17–20^ However, the massive applications of *n*-alkanes in solar energy utilization fields are seriously restricted by liquid leakage problem during phase transition. Encapsulating the *n*-alkanes to form core–shell microcapsules is considered an effective method. However, the encapsulation process is always complex and the phase change enthalpy of the encapsulated PCMs significantly decreased.^21–23^ Therefore, it is urgently needed to fabricate *n*-alkane containing PCMs with high phase change enthalpy, shape and thermal stability.

Recently, extensive attention has been paid to impregnating PCMs in three-dimensional (3D) aerogels to construct shape-stable, leak-proof PCM composites.^24–26^ Especially, nanofibrillated cellulose (NFC) aerogel not only can effectively prevent leakage of solid–liquid PCMs, but also are environmental-friendly. As a result, it is necessary to study the solid–liquid phase change material with NFC aerogel as the supporting material. Kim *et al.*^27^ prepared carbon foam by using methyl cellulose (CMC). In addition, the composite PCMs (CPCMs) incorporate erythritol into the cellulose carbon foam by vacuum impregnation. The thermal cycling test indicated the CPCMs exhibited much less phase change enthalpy loss than the pure erythritol. These results likely occurred because the pores of carbon foam can prevent the leakage of erythritol, thus minimizing the latent heat loss during thermal cycling tests through capillary forces. Lei *et al.*^28^ prepared a novel CPCMs by impregnating PEG in NFC/boron nitride nanosheet (BNNS) aerogels. The CPCMs could also keep their original shape at a 101 °C. In addition, the CPCMs with a relatively small loading of BNNSs could also maintain a high-level of latent heat (136.8 J g^−1^). These studies indicate that NFC aerogel can effectively encapsulate solid–liquid phase change materials. However, it is obvious that the CPCMs have low solar energy utilization efficiency.

Therefore, it is necessary to exploit the solid–liquid phase change material with NFC aerogel as the support material. At the same time, the CPCMs have high solar energy utilization efficiency. PDA is regarded as a photothermal conversion material, which can not only be used in near-infrared photothermal therapy of cancer, but also exert high photothermal effect in broad-spectrum of sunlight.^29–31^ Cao *et al.*^32^ prepared an efficient bilayer photothermal film based on hydroxyapatite (Ca_10_(PO_4_)_6_(OH)_2_, HA) nanowires. Its top layer comprises polydopamine (PDA)-coated HA (HA@PDA) nanowires, which can effectively convert solar energy to heat. Besides, the bottom layer comprises chitosan (CS)-bonded HA nanowires (HA–CS), which can be used as a thermal insulator. This double-layer photothermal film has excellent light absorption, thermal insulation, porosity and stability.

In our work, NFC/PDA suspensions were synthesized by an *in situ* self-polymerization of PDA in NFC suspensions. Then, the hybrid gel was prepared by using cation-induced gelation of aluminum ions, which was freeze-dried to form the NFC/PDA hybrid aerogel. Finally, the CPCMs were prepared by vacuum impregnation of molten *n*-octacosane. The microstructure, phase change property, thermal reliability, shape stability, crystalline property, storage efficiency and photothermal conversion of CPCMs were systematically investigated. As previously envisioned, CPCMs with the addition of PDA exhibited a significant increase in photothermal conversion efficiency, as well as high energy storage capacity, excellent thermal cycle stability, good shape stability and thermal stability. The results above indicated the great potential of our synthesized CPCMs for solar energy utilization.

## Experimental

### Materials

NFC (length: 1500–3500 nm, diameter: 4–15 nm, carboxyl content: 2 mmol g^−1^) was supplied from Guilin Qihong Technology Co., Ltd., China. *n*-Octacosane (AR) and DA·HCl (AR) were purchased from Chengdu Huaxia Chemical Reagent Co., Ltd., China. Aluminum nitrate nonahydrate (Al(NO_3_)_3_·9H_2_O, AR) was purchased from Chengdu Kelong Chemical Reagent Co., Ltd. China. Tris(hydroxymethyl)aminomethane (Tris, AR) was purchased from Aladdin Reagent Co., Ltd., China.

### Preparation of NFC/PDA solution

**Scheme 1:**
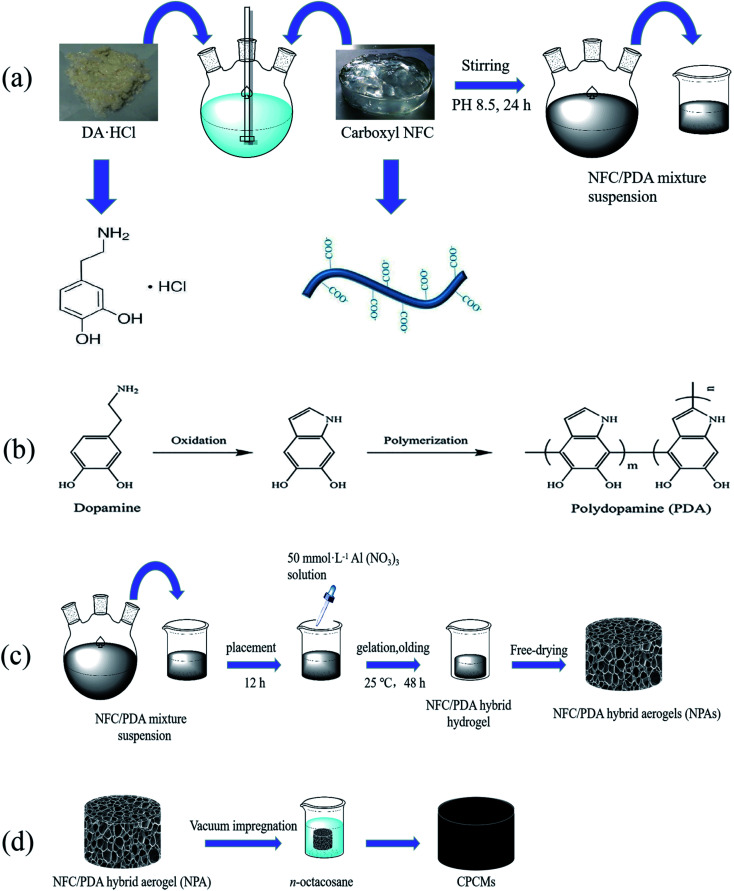
Schematic diagram of the synthesis steps of CPCMs. (a) Synthesis process of NFC/PDA solution, (b) synthesis mechanism of PDA, (c) synthesis process of NPAs, (d) synthesis process of CPCMs.

The NFC/PDA solution was synthesized by an *in situ* self-polymerization of PDA on the surface of NFC. Scheme 1a shows the procedure used to prepare the NFC/PDA solution, 20 g of NFC suspension (1 wt%) was poured into a triple flask. Then, the suspension was adjusted to pH 8.5 by Tris DA·HCl was added at 0%, 12.5%, 25%, 50%, 100%, 200% of the mass of cellulose, respectively. After stirred for 24 h, various ratios of NFC/PDA solutions were obtained. The reaction process was shown in Scheme 1b.

### Preparation of NFC/PDA hybrid aerogels (NPAs)

NFC/PDA hybrid aerogels (NPAs) were fabricated by using cation-induced gelation of a suspension of NFC/PDA solution, followed by freeze-drying. As shown in Scheme 1c, 20 g of the NFC/PDA solution was poured into a beaker, follow by placing it for 12 h to smooth the surface. A 50 mmol L^−1^ Al(NO_3_)_3_ solution was prepared, and the Al(NO_3_)_3_ solution was added dropwise into the NFC/PDA solution along the wall of the beaker, then the beaker was gelatinized and aged at 25 °C for 48 h. The aged gels were pre-freezed in the refrigerator for 12 h and subsequently dried in a freeze dryer for 48 h to obtain various hybrid aerogels. Hybrid aerogels named NPA-P0, NPA-P12.5, NPA-P25, NPA-P50, NPA-P100, NPA-P200 corresponded to 0%, 12.5%, 25%, 50%, 100%, 200% of the added DA relative to the cellulose mass, respectively.

### Preparation of phase change composites (CPCMs)

The CPCMs were prepared by directly immerging NPAs into a glass beaker containing molten *n*-octacosane. The synthesis process was shown in Scheme 1d. Firstly, the *n*-octacosane powder was added into the beaker, and then it completely melted into liquid at 80 °C. The hybrid aerogel was immersed into the liquid, and the beaker was put into a vacuum oven at 80 °C to remove the gas in the hybrid aerogel. The CPCMs prepared by using NPA-P0, NPA-P12.5, NPA-P25, NPA-P50, NPA-P100, NPA-P200 were named CPCM-P0, CPCM-P12.5, CPCM-P25, CPCM-P50, CPCM-P100, CPCM-P200 respectively. The loading rate (*R*_L_) of *n*-octacosane in CPCMs was calculated by the following equation:
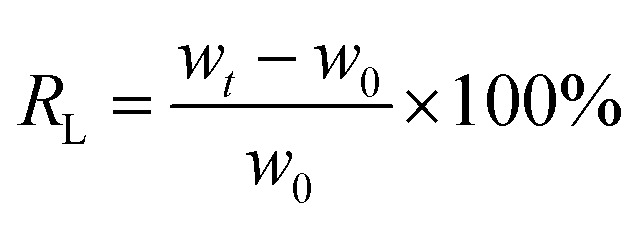
where *w*_*t*_ is the weight of CPCMs, *w*_0_ is the weight of the aerogels.

### Characterization

The chemical structure of *n*-octacosane, PDA, NFC aerogel and CPCMs were analysed by an FTIR spectrometer (Nicolet NEXUS-670, America). The wave number range is 4000–400 cm^−1^, and the resolution is 4 cm^−1^. The photoelectron spectra of the NPAs were measured using an X-ray photoelectron spectroscopy (XPS) (K-Alpha^+^, Thermo Scientific, USA). The crystallization property of *n*-octacosane, NFC, PDA, CPCMs were determined by X-ray power diffraction (XRD, bruke discover) with a 2*θ* range from 5–50° and a scanning rate of 10° min^−1^. The shape stability of CPCMs was measured by a heating platform-digital camera. The samples were placed in a heating platform at 100 °C, followed by taking with a digital camera to record the morphological changes. JEM-7500F scanning electron microscope (SEM, JEOL, Japan) were employed to measure the morphology of NPAs and CPCMs.

The thermogravimetric analyser (TG, Netzsch STA409PC, Germany) was used to evaluate the thermal stability of CPCMs. The samples were measured with a temperature change of 10 °C min^−1^ under N_2_ atmosphere. The sample mass is 4–8 mg, and the temperature program is increased from 50 to 700 °C. The melting and crystallization processes of CPCMs were measured by a differential scanning calorimeter (DSC, PerkinElmer DSC8500, America). Samples were measured at a temperature variation of 10 °C min^−1^ under N_2_ atmosphere. The sample mass is 3–5 mg, and the temperature program is from 10 to 100 °C. The thermal reliability of CPCMs was evaluated by accelerated melting/freezing cycling test. The thermal property of the samples after 100 times cycles between −10 and 100 °C were studied by DSC.

The photothermal conversion performance of CPCMs were measured by using the simulated xenon lamp system (CEL-S500). The xenon lamp operating voltage is 20 V and the working current is 15–25 A. The samples were connected to thermocouples and measured in a sealed environment under a xenon lamp.

## Results and discussion

### FT-IR analysis of CPCMs

**Fig. 1:**
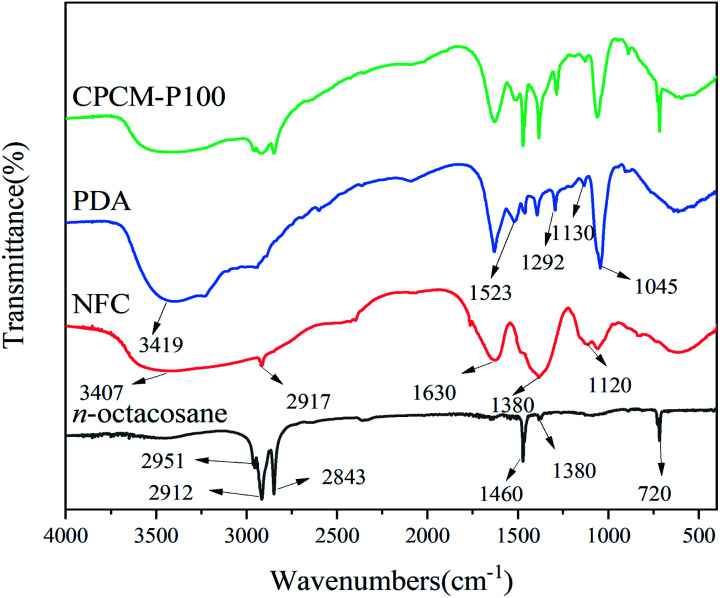
FTIR spectra of *n*-octacosane, NFC, PDA, CPCM-P100.

In this work, the CPCMs were prepared by impregnating NPAs in a glass beaker containing molten *n*-octacosane. Fig. 1 shows the FTIR spectra of *n*-octacosane, NFC, and PDA. In the *n*-octacosane spectrum, the characteristic peak at 720 cm^−1^ was attributed to the horizontal rocking vibration of (–CH_2_–)_*n*_ (*n* ≥ 4),^33^ and the characteristic peaks at 1380, 1460, 2951 cm^−1^ corresponded to the stretching vibration of –CH_3_, while the characteristic peaks at 2843 and 2912 cm^−1^ corresponded to the stretching vibration of –CH_2_–. For NFC, the characteristic peaks at 1120, 1630, and 3407 cm^−1^ corresponded to the C–O–C vibration in the six-membered ring,^34^ –COO–, –OH stretching vibration, respectively, while the characteristic peaks at 1380 and 2917 cm^−1^ were attributed to the C–H stretching vibration. In the PDA spectrum, the characteristic peaks at 1130, 1292, and 1523 cm^−1^ corresponded to the stretching vibration of C–O, the stretching vibration of C–N, the bending vibration of N–H, respectively. The characteristic peaks at 1045 and 3419 cm^−1^ were attributed to bending vibration and stretching vibration of –OH, respectively. The FTIR peaks of *n*-octacosane, NFC and PDA appeared in CPCM-100 spectrum, which proved that there was only physical interaction between the three components, and the CPCMs were successfully synthesized.

### XPS analysis of NPAs

**Fig. 2:**
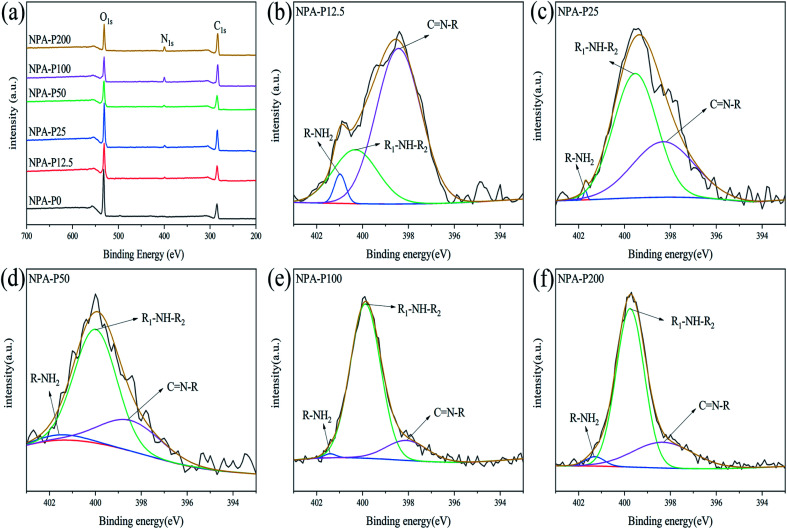
(a) XPS spectra of NPAs, N_1s_ high-resolution spectrum of (b) NPA-P12.5, (c) NPA-P25 (d) NPA-P50 (e) NPA-P100 (f) NPA-P200.

We further conducted element type and valence of obtained NPAs. As shown in Fig. 2a, for NPA-P0, the signals around 286 eV and 531 eV were attributed to C_1s_ and O_1s_, respectively.^35^ As for NPA-P12.5, NPA25, NPA-P50, NPA-P100, NPA-P200, not only the signals of C_1s_ and O_1s_ appeared at 286 eV and 531 eV, but also the peak of N_1s_ appeared at 400 eV. XPS results indicated the successful synthesis of PDA. As shown in Fig. 2b–f, the signal of N_1s_ could be divided into three peaks: C=N–R (398.1 eV), R_1_–NH–R_2_ (399.9 eV), R–NH_2_ (401.4 eV),^35^ the peak area of secondary amine (R_1_–NH–R_2_, 399.9 eV) was the largest except for NPA-P12.5. It was proved that the content of R_1_–NH–R_2_ was the highest, which accorded with the structure of PDA (Scheme 1d). Table 1 summarizes the nitrogen-to-carbon (N/C) ratio and N_1s_ XPS data of NPAs. The overall trend of N/C increased with the addition of PDA. The N_1s_ specific data again showed the highest content of R_1_–NH–R_2_. The XPS data further demonstrated the successful synthesized of NPAs.

**Table 1:** XPS data of the NPAs

Sample	N/C	High-resolution N_1s_ components (%)		
		N1 area (%), 398.1 eV, C=N–R	N2 area (%), 399.9 eV, R_1_–NH–R_2_	N3 area (%), 401.4 eV, R–NH_2_
NPA-P0	0	—	—	—
NPA-P12.5	0.063	71.73	24.83	3.44
NPA-P25	0.065	38.71	60.94	0.35
NPA-P50	0.121	27.03	69.56	3.41
NPA-P100	0.333	14.94	84.25	0.81
NPA-P200	0.273	25.20	71.92	2.88

### Morphology of NPAs and CPCMs

**Fig. 3:**
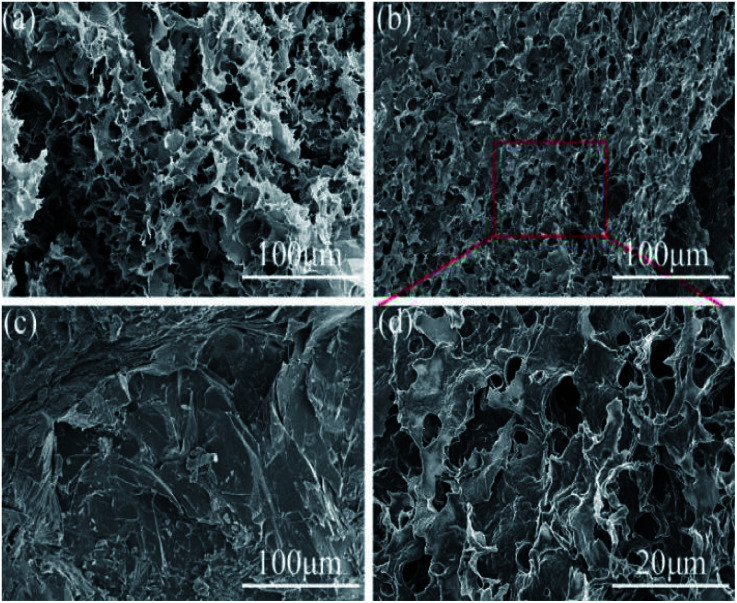
SEM images of NPAs and CPCMs: (a) NPA-P0, (b, d) NPA-P100, (c) CPCM-P100.

SEM was employed to investigate the morphology of the various aerogels and composite phase change materials. The cross-sectional morphology of the NFC aerogel was shown in Fig. 3a, it could be observed that a network-like interconnected porous structure was formed in the NFC aerogel. The pore size was about a few micrometers, which was provided with capillary force that could effectively prevent the leakage of solid–liquid phase change materials. As shown in Fig. 3b, a mesh interconnected porous structure was also formed in the hybridized aerogel of NFC/PDA. Local magnification analysis as presented in Fig. 3d showed that a fold formed on the surface of the pore wall. It was indicated that the PDA had been homogeneously distributed in the NFC aerogel. After impregnation with *n*-octacosane, the cross-sectional morphology of CPCM-P100 was shown in Fig. 3c, the mesh-like porous structure of CPCM-P100 had completely disappeared, and a sheet-like tight structure appeared. NPA-P100 and *n*-octacosane had no obvious interface, which proved the excellent compatibility between *n*-octacosane and NPA-P100. The capillary force and good affinity of the porous aerogel not only allowed NPA-P100 to absorb more *n*-octacosane, but also effectively prevented the leakage when the phase change occurred.

### Loading rates of *n*-octacosane for CPCMs

**Fig. 4:**
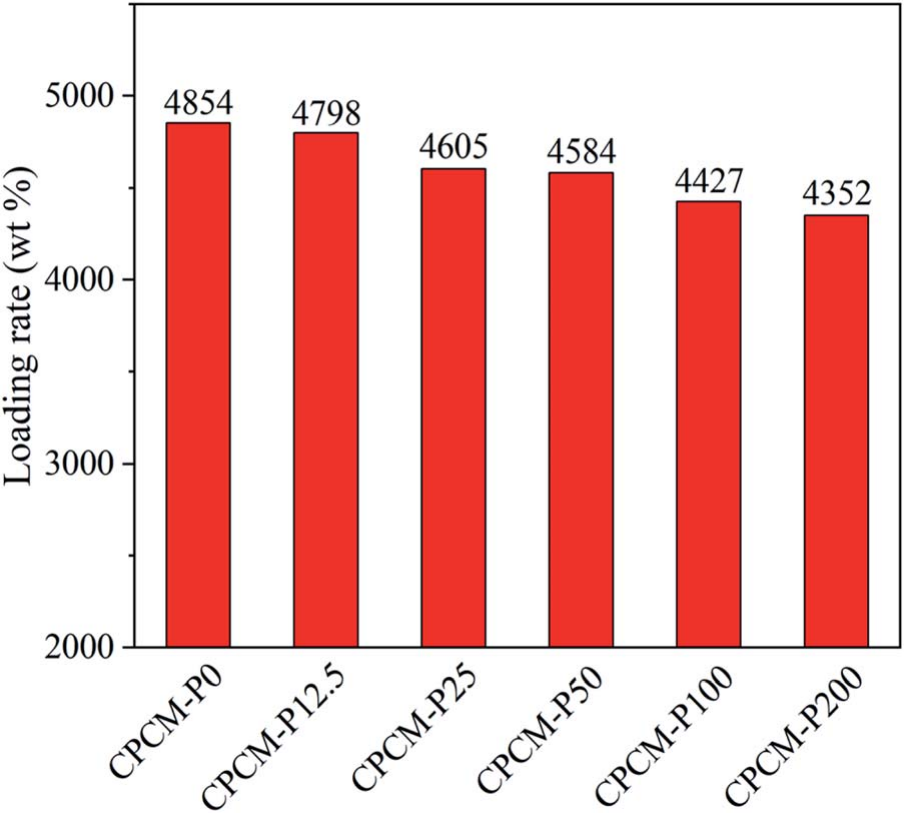
Loading rates of *n*-octacosane for CPCMs.

The loading ratio (*R*_L_) of *n*-octacosane in CPCMs was calculated to evaluate the energy storage capacity of CPCMs. As shown in Fig. 4, the RL of CPCM-P0 was as high as 4854%, which was attributed to the high porosity and pore volume characteristic of NFC aerogels. With the increase of PDA content, the RL of CPCMs decreased gradually, and the loading rate of CPCM-P200 was still as high as 4352%. Such a high RL still allowed CPCMs to maintain a very high energy storage capacity.

### Form-stability of CPCMs

**Fig. 5:**
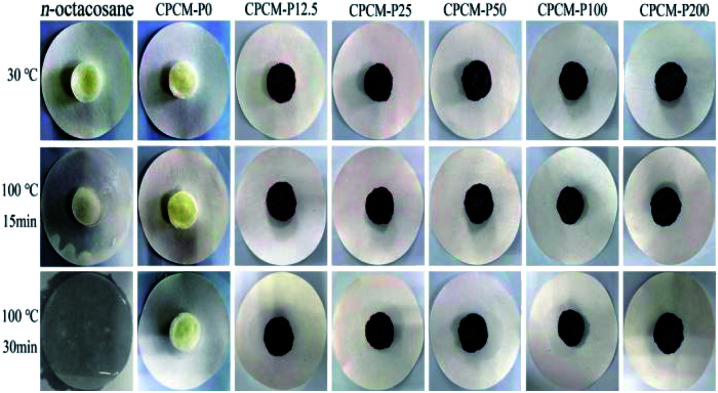
Digital photographs of *n*-octacosane, CPCMs at 100 °C.

Shape thermal stability is critical for practical applications of solid–liquid phase change materials. *N*-Octacosane, CPCMs were placed in a heating platform (100 °C) and a digital camera was used to take pictures to evaluate the shape thermal stability of the CPCMs. As shown in Fig. 5, all samples were solid at room temperature. After heating at 100 °C for 15 minutes, *n*-octacosane had mostly melted, while CPCMs showed no signs of leakage. When heating at 100 °C for 30 minutes, *n*-octacosane had completely melted into a clear liquid, while only slight leakage of CPCMs was absorbed by filter paper. The leakage tests showed that the capillary force of aerogel could effectively prevent liquid leakage. The CPCMs exhibited ideal shape stability.

### Thermal properties of CPCMs

**Fig. 6:**
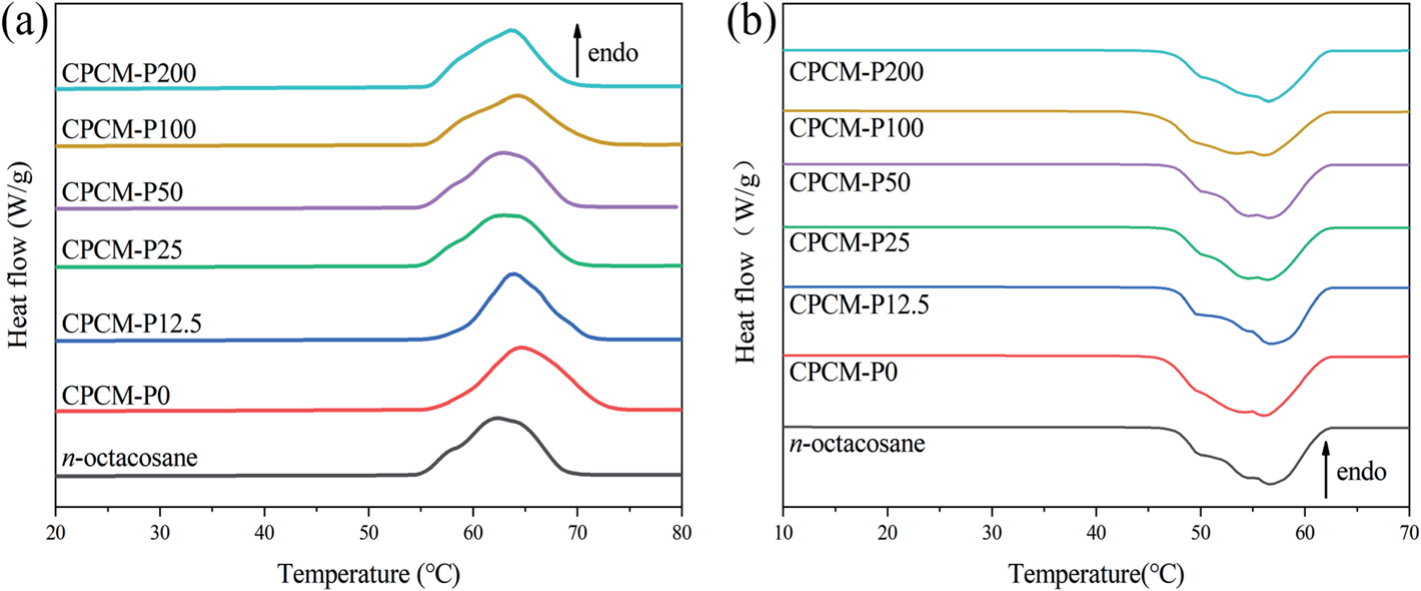
DSC curves of *n*-octacosane and CPCMs: (a) melting process, (b) freezing process.

Photothermal conversion materials with high thermal storage capacity and suitable phase change temperature are vital for practical application. The thermal storage capacity of CPCMs was analyzed by DSC. The DSC curves of *n*-octacosane, CPCMs were shown in Fig. 6. Melting point (*T*_m_), freezing point (*T*_f_), melting enthalpies (Δ*H*_m_), and freezing enthalpies (Δ*H*_f_) were listed in the Table 2. As shown in Fig. 6a and b, the exothermic and endothermic curves of CPCMs were very similar to *n*-octacosane, which indicated that NPAs did not react chemically with *n*-octacosane and CPCMs retained excellent phase change properties. As shown in Table 2, the *T*_m_ and *T*_f_ of *n*-octacosane were lower than CPCMs, which were attributed to the porous structure of the aerogel that impeded the movement of *n*-octacosane molecules during melting and freezing. The Δ*H*_m_ of CPCMs from 249.8 to 263.1 J g^−1^ and the Δ*H*_f_ from 248.5 to 262.1 J g^−1^, there was only a slight decrease compared to the thermal storage capacity of *n*-octacosane, which proved that the synthesized CPCMs had excellent energy storage capacity. The comparison in Δ*H*_m_ between CPCMs in this study and other reported CPCMs were shown in Table 3. The DSC results indicated that the synthesized CPCMs possessed satisfactory TES density (>248 J g^−1^) and suitable phase change temperature for the efficient utilization of solar energy.

**Table 2:** Thermal storage capacity of *n*-octacosane and CPCMs

Sample	*T*_m_ (°C)	Δ*H*_m_ (J g^−1^)	*T*_f_ (°C)	Δ*H*_f_ (J g^−1^)
*n*-Octacosane	62.4	264.7	55.1	263.4
CPCM-P0	64.4	260.2	56.4	258.8
CPCM-P12.5	64.1	263.1	56.9	262.1
CPCM-P25	64.0	254.4	56.5	252.8
CPCM-P50	63.8	256.1	56.3	254.7
CPCM-P100	64.3	250.4	56.4	248.3
CPCM-P200	64.5	249.8	56.5	248.5

**Table 3:** The comparison in Δ*H*_m_ between CPCMs in this study and other reported composite PCMs

PCMs	Δ*H*_m_ (J g^−1^)	Ref.
Carbon nanotube-decorated binary-core phase-change microcapsules	187.9	36
Magnetic cellulose nanocrystals hybrids reinforced phase change fiber composites	83.1	37
Leakage-proof microencapsulation of phase change materials by emulsification with acetylated cellulose nanofibrils	173.0	38
Constructing cellulose nanocrystal/graphene nanoplatelet networks in phase change materials	145.5	39
Temperature and pH dual-stimuli-responsive phase-change microcapsules	160.0	40
Highly efficient photothermal conversion capric acid phase change microcapsule	92.06	41
Three-dimensional hierarchical porous graphene foam-carbon nanotube hybrid structure phase change materials composites	140.48	42
Polyethylene glycol (PEG)/poly acrylic acid (PAA)/silica (SiO_2_) composite phase change materials	172.9	43
Highly thermally conductive and flexible phase change composites enabled by polymer/graphite nanoplatelet-based dual networks	169.5	44
MXene-wrapped bio-based pomelo peel foam/polyethylene glycol composite phase change material	156.1	45
This work	262.1	—

### Crystalline properties of CPCMs

**Fig. 7:**
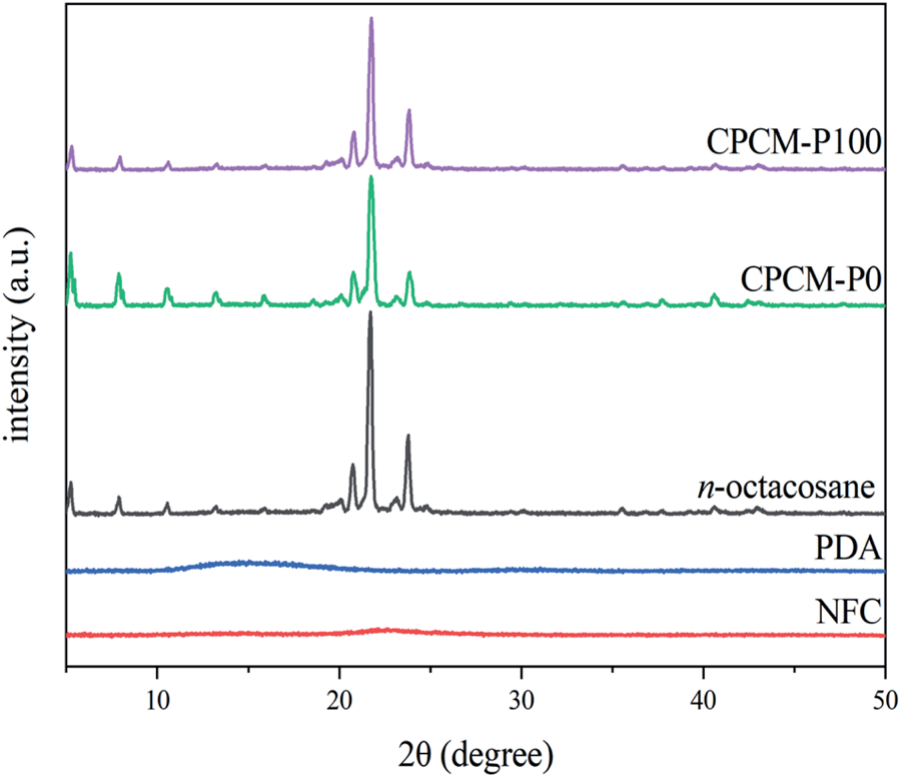
XRD patterns of NFC, PDA, *n*-octacosane, CPCM-P0, and CPCMP100.

The phase change behavior of CPCMs is strongly affected by the crystallization properties. Therefore, the crystallization properties of NFC, PDA, *n*-octacosane, CPCM-P0, and CPCM-P100 were further analyzed using XRD. As shown in Fig. 7, *n*-octacosane displayed thermodynamically stable triple oblique crystalline, evidenced by that its diffraction peaks appear at 20.13°, 20.79°, 21.69°, and 23.77° correspond to the (010), (011), (100), and (111) planes of triple oblique crystalline, respectively.^46^ The XRD curves of NFC and PDA showed a broad peak at 22.7° and 15.1°, respectively, proving that both NFC and PDA were amorphous materials. The positions of the diffraction peaks in XRD curves of CPCM-P0 and CPCM-P100 were consistent with *n*-octacosane, while the intensity of diffraction peaks weakened slightly. It proved that the *n*-octacosane crystallization process in CPCM-P0 and CPCM-P100 was bound by the porous structure of aerogels, which was consistent with the increase of melting point and crystallization temperature in DSC curves. Although the crystallization behavior of *n*-octacosane in CPCMs is limited, CPCMs still had excellent crystallization ability.

### Reversibility of CPCMs

**Fig. 8:**
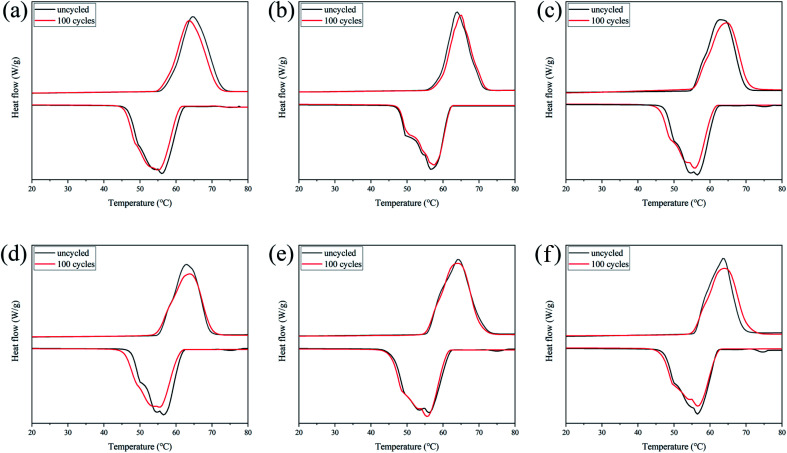
DSC curves of the CPCMs after 100 melting–freezing cycles: (a) CPCM-P0, (b) CPCM-P12.5, (c) CPCM-P25, (d) CPCM-P50, (e) CPCM-P100, (f) CPCM-P200.

It is important that the thermal properties of PCMs remain stable after long-term use. Therefore, CPCM-P0, CPCM-P12.5, CPCM-P25, CPCM-P50, CPCM-P100, CPCM-P200 were placed on a heating platform for melting/freezing cycles lasting for 100 times, then their thermal property was tested by DSC. The measurement results were shown in Fig. 8. The DSC curves of CPCM-P0, CPCM-P12.5, CPCM-P25, CPCM-P50, CPCM-P100, and CPCM-P200 show little change in the shape of melting and freezing peaks, indicating that the phase change properties of CPCM-P0, CPCM-P12.5, CPCM-P25, CPCM-P50, CPCM-P100, and CPCM-P200 without any change in the phase change properties. The specific datas were shown in Table 4, after 100 melting–freezing cycles, the change in *T*_m_ and *T*_f_ did not exceed 0.5 °C, the change in Δ*H*_m_ and Δ*H*_f_ did not exceed 2 J g^−1^, which fully proved that the synthesized CPCMs possessed excellent thermal cycling stability.

**Table 4:** Thermal storage capacity of *n*-octacosane and CPCMs after 100 melting–freezing cycles

Sample	*T*_m_ (°C)	Δ*H*_m_ (J g^−1^)	*T*_f_ (°C)	Δ*H*_f_ (J g^−1^)
CPCM-P0	64.0	257.4	56.1	257.7
CPCM-P12.5	63.9	261.8	56.8	260.4
CPCM-P25	64.3	252.1	57.0	250.4
CPCM-P50	63.9	253.3	56.5	253.8
CPCM-P100	64.3	250.3	56.2	248.4
CPCM-P200	64.7	249.4	56.3	248.7

### Thermal stability of CPCMs

**Fig. 9:**
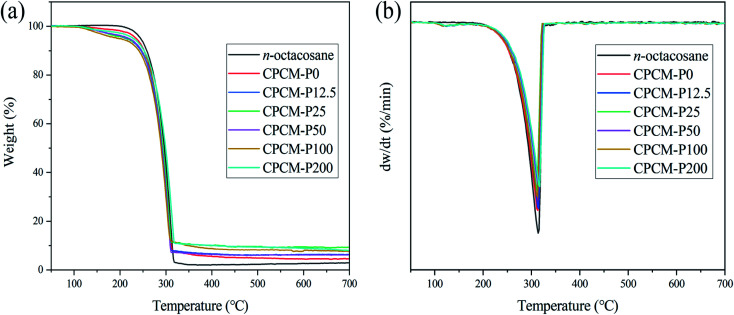
TG and DTG curves of *n*-octacosane and CPCMs: (a) TG, (b) DTG.

The thermal stability of CPCMs is crucial for practical applications of PCMs, and the thermal stability of the samples were measured by TG. As shown in Fig. 9a and b, *n*-octacosane exhibits a one-step thermal decomposition behavior at temperatures ranging from 223 to 325 °C, with a maximum weight loss rate temperature (*T*_max_) of 313 °C, which was mainly because of the volatilization of straight-chain alkane molecules.^47^ For the CPCMs samples, the first stage of weight loss occurred from 113 °C to 194 °C in Fig. 9a, which were attributed to the existence of some moisture in the samples due to the strong water absorption of NFC. As shown in Fig. 9b, the *T*_max_ for CPCM-P0, CPCM-P12.5, CPCM-P25, CPCM-P50, CPCM-P100, and CPCM-P200 were 312.8, 314.5, 312.0, 315.0, 312.4 and 316.9 °C, respectively. The thermal decomposition process of the synthesized CPCMs were similar to that of *n*-octacosane, which once again proved that NFC/PDA aerogel did not chemically react with phase change materials, and CPCMs had excellent phase change properties. The decomposition of CPCMs above 220 °C was much higher than the temperature of the sample during photothermal conversion, indicating that the synthesized CPCMs were a material with ideal thermal stability in its application range.

### Solar-thermal conversion and storage of CPCMs

**Fig. 10:**
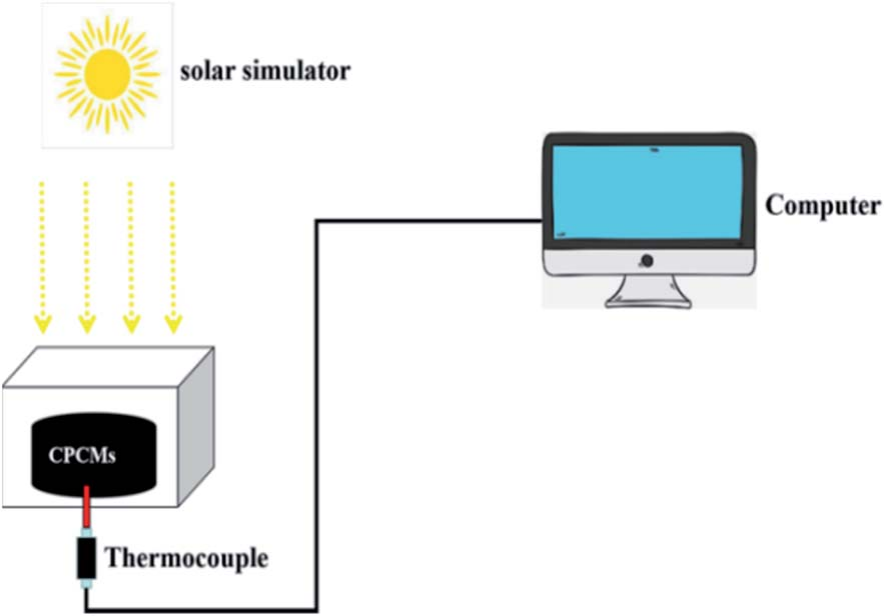
Schematic diagram of photothermal conversion measurement system.

In order to evaluate the photothermal conversion performance of CPCMs, a test apparatus was designed as shown in Fig. 10. Using a xenon lamp to simulate sunlight, the radiation intensity was 250 mW cm^−2^, and the CPCMs sample were a cylinder with a diameter of 3 cm and a height of 0.75 cm. A thermocouple was inserted into the sample of CPCMs, and an external computer was used to record the data. The solar thermal conversion and energy storage efficiency (*η*) of CPCMs could be calculated by the following equation:
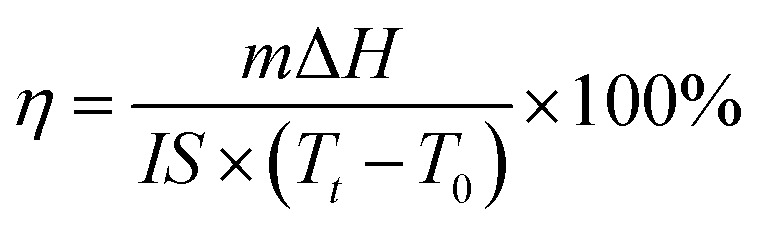
where *m* represents the weight of the sample, Δ*H* represents the melting enthalpy of the sample. *I* re the radiation intensity. *S* represents the received radiation area of the sample. *T*_*t*_ and *T*_0_ represents the end time and the start time of the sample phase change, respectively.^48^

**Fig. 11:**
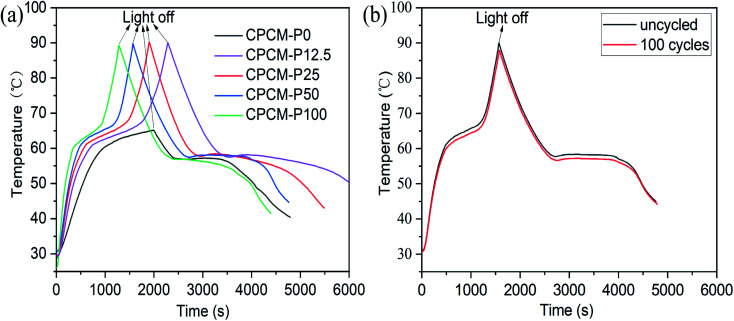
Temperature–time curves of CPCM under light irradiation at 250 mW cm^−2^: (a) CPCMs, (b) CPCM-P50 after 100 light on and off cycles.

The temperature curves of the CPCMs with different PDA contents are shown in Fig. 11a. Under the sunlight, the temperature of CPCMs rises rapidly and reached a temperature plateau (61–67 °C), which indicated that the material underwent a solid–liquid phase transition as latent heat, and the heat energy converted by light energy was stored. When the sunlight was stopped, the temperature of CPCMs decreased rapidly and a temperature plateau appeared (57–55 °C), which was due to the cooling crystallization and exotherm of *n*-octacosane in CPCMs. In addition, with the increased of PDA content, the time to reach the melting platform became shorter, and the temperature range of the platform becomes narrower. This is mainly because PDA in CPCMs could effectively convert light energy into heat energy. The light-thermal conversion and storage efficiency of CPCMs were calculated with *η* values of 47.1%, 58.4%, 65.4, 74.9% and 86.7% for CPCM-P0, CPCM-P12.5, CPCM-P25, CPCM-P50, and CPCM-P100 respectively, demonstrating that PDAs could efficiently convert light energy into heat energy.

The repeatability test of solar thermal conversion and energy storage is necessary for the practical application of CPCM. The CPCM-P50 was carried out 100 light on and off cycles. The measurement results were shown in Fig. 11b. After 100 cycles, the photothermal conversion efficiency and energy storage efficiency of CPCM-P50 were still as high as 72.1%, which was only 2.8% lower than that of the uncycled CPCM-P50. The temperature–time curves indicated that CPCMs had excellent photothermal cycle stability.

## Conclusion

In our study, we first self-polymerization PDA in an NFC suspension to form a stable solution, the hybrid gel was prepared by using cation-induced gelation of aluminum ions, which was freeze-dried to form NPAs. NPAs were impregnated in a vacuum environment to introduce *n*-octacosane to synthesize CPCMs. The NPAs with three-dimensional (3D) interconnected porous structures well supported the *n*-octacosane within the nanostructural frameworks and effectively prevented the leakage and diffusion of *n*-octacosane above its melting point. In CPCMs, the PDA acted as a light energy trap, which converted the absorbed solar energy into heat energy. The *n*-octadecane acts as a thermal energy storage vessel that absorbs thermal energy as latent heat. The photothermal conversion tests showed that the addition of PDA effectively improved the solar thermal conversion efficiency and storage efficiency. CPCM-P100 increased the *η* from 47.3 to 86.7% compared to CPCM-P0. DSC tested showed that CPCMs possessed an extremely high phase change enthalpy (>248 J g^−1^) and excellent thermal reliability. After 100 melting–freezing cycles, the change in *T*_m_ and *T*_f_ did not exceed 0.5 °C, and the change in Δ*H*_m_ and Δ*H*_f_ did not exceed 2 J g^−1^. In addition, leakage tests and TG tests showed that CPCMs had excellent shape stability and thermal stability. These excellent properties showed that CPCMs was an ideal material for solar energy utilization.

## Conflicts of interest

The authors declare no conflict of interest.

## Supplementary Material
